# Supplementary material for: Biotope-dependent resistance to reactive oxygen species, antibiotic tolerance, and virulence of Staphylococcus aureus
Source: Commun Biol. 2026 Oct 1;9:1192. doi: 10.1038/s42003-026-10771-2 (PMC13631261; doi:10.1038/s42003-026-10771-2)
Supplement: Supplementary file 2 [file 42003_2026_10771_MOESM2_ESM.pdf]

Description of Additional Supplementary Files

File name: Supplementary Data 1
Description: Proteomics data table shows quantitative information of proteins quantified in 4/5 biological replicates per group. These proteins, which are represented in the Venn Diagram, were used for downstream proteomic analysis. The table includes MaxQuant outputs such as: UniProt ID, annotated name, number of peptides, MW, and normalized intensity values.

File name: Supplementary Data 2
Description:

1. Quantitative proteomics data of proteins related to fatty acid synthesis that was extracted for volcano plot analysis. Proteomics data table shows quantitative information of proteins quantified in 4/5 biological replicates per group. These proteins, which are represented in the Venn Diagram, were used for downstream proteomic analysis. The table includes MaxQuant outputs such as: UniProt ID, annotated name, number of peptides, MW, and normalized intensity values.
2. Quantitative proteomics data of proteins related to the oxidative stress response that was extracted for volcano plot analysis. Proteomics data table shows quantitative information of proteins quantified in 4/5 biological replicates per group. These proteins, which are represented in the Venn Diagram, were used for downstream proteomic analysis. The table includes MaxQuant outputs such as: UniProt ID, annotated name, number of peptides, MW, and normalized intensity values.
3. Quantitative proteomics data of proteins related to virulence that was extracted for volcano plot analysis. Proteomics data table shows quantitative information of proteins quantified in 4/5 biological replicates per group. These proteins, which are represented in the Venn Diagram, were used for downstream proteomic analysis. The table includes MaxQuant outputs such as: UniProt ID, annotated name, number of peptides, MW, and normalized intensity values.

File name: Supplementary Data 3
Description: Source data.
